# Acute and Subacute Toxicological Evaluation of the Aerial Extract of* Monsonia angustifolia* E. Mey. ex. A. Rich in Wistar Rats

**DOI:** 10.1155/2016/4952485

**Published:** 2016-09-08

**Authors:** Anthony Jide Afolayan, Olubunmi Abosede Wintola, Gerda Fouche

**Affiliations:** ^1^Medicinal Plants and Economic Development Research Centre (MPED), Department of Botany, University of Fort Hare, Eastern Cape, South Africa; ^2^Council for Scientific and Industrial Research (CSIR), Pretoria, South Africa

## Abstract

The acute and subacute toxicity profile of the aerial extract of* Monsonia angustifolia* in Wistar rats was evaluated. The Organization for Economic Cooperation and Development (OECD) 420 guideline was adopted in the acute toxicity testing with a single oral dose of 5000 mg/kg (b.w.). For the 28-day daily oral dosing, the extract was administered at 75, 150, and 300 mg/kg b.w.; 1% ethanol in sterile distilled water was used as control. Clinical toxicity signs were subsequently evaluated. At a single dose of 5000 mg/kg b.w. the extract elicited no treatment-related signs of toxicity in the animals during the 14 days of experimental period. In the subacute toxicity, there was no significant difference in hematological, renal, and liver function indices. However, dose-dependent significant increases were observed on the plasma concentrations of white blood cell and platelet counts of the treated animals compared to the control group. While cage observations revealed no treatment-facilitated signs of toxicity, histopathological examinations of the kidneys and liver also showed no obvious lesions and morphological changes. These results suggest that the extract may be labelled and classified as safe and practically nontoxic within the doses and period of investigation in this study.

## 1. Introduction

The genus* Monsonia* is dispersed over both hemispheres in Africa, America, Europe, Asia, and Australia [1]. It is widespread in Africa from Nigeria to Somalia and in the south in South Africa and also in Madagascar [2].* Monsonia angustifolia* E. Mey. ex. A. Rich, commonly known as Crane's bill, Alsbos, Angelbossie, or Teebossie, belongs to the family Geraniaceae. It is a suberect annual plant growing in sandy soils, usually on granite, often on rocky areas, and along roadsides. Teebossie is widely distributed in the southern African region, from South Africa to Lesotho, Swaziland, Namibia, and Mozambique.* Monsonia angustifolia* (MA) is provincially distributed in the Eastern Cape, Free State, Gauteng, KwaZulu-Natal, Limpopo, and Mpumalanga in South Africa [3]. Its red thick woody based stems are usually about 50 cm high with characteristic short pubescent gland-tipped hairs. The leaves are narrow with irregular tooth of cuneate based oblong or elliptic margins. While its flowers are small and purple-tinted with overlapping toothed petals and the stamens are arranged in an outward spreading pattern from the center, the fruits are held in an erect fashion and are approximately 50–90 mm long. MA is rich in calcium, iron, proteins, and vitamin C and the presence of these constituents in its organs may be attributed to its excellently displayed importance as a medicinal agent [4]. Ethnomedicinally, MA has therapeutic significance as a blood cleanser and aphrodisiac and enhances libido [5]. Fouche et al. [6] have also reported its sexual competence enhancing ability in rats. Its pharmacological relevance in the treatment of heartburn, anthrax, and diarrhoea has also been documented [7].

The ethnomedicinal application of different formulations of MA to treat various diseases is increasing appreciably. Despite the profound therapeutic significance of MA, there is a paucity of information in the literatures about the safety of its standardized aqueous extract. Accordingly, since nontoxicity is one of the criteria set by the World Health Organization for the use of herbs as medicines, the present study was therefore conceptualized to investigate the effect of oral acute and 28-day repeated dose administration of standardized extract of MA on haematological and kidney and liver function parameters of Wistar rats.

## 2. Experimental

### 2.1. Chemicals, Reagents, and Assay Kits

The assay kits for the determination of alanine aminotransferase (ALT) and aspartate aminotransferase (AST) were supplied by Randox Laboratories Ltd., United Kingdom, while those of alkaline phosphatase (ALP) and gamma glutamyl transferase (GGT) were obtained from Roche Diagnostics GmbH, Mannheim, Germany. All other chemicals and reagents were of analytical grade and prepared in glass-distilled water.

### 2.2. Plant Collection, Authentication, and Processing

Fresh plant materials comprising the aerial parts of* Monsonia angustifolia* were collected near Chuenespoort, Polokwane area, Limpopo Province, South Africa. The plant was identified at the South African Biodiversity Institute (SANBI), where a voucher specimen (number 582251.0) was prepared and deposited. The identified sample was thoroughly rinsed under running water to remove foliar contaminants, air-dried to constant weight, and subsequently pulverized in a hammer mill.

### 2.3. Extract Preparation

The aqueous extract was prepared by a method similar to making a tea infusion. One liter of deionized water was added to 86 g of the ground material and boiled for an hour. The water solution was left to cool to room temperature. The resulting infusion was filtered and subsequently freeze-dried. This yielded a brownish fluffy powder corresponding to 22.16 g of the crude extract. The crude extract obtained was thereafter standardized by dissolution in 200 mL of distilled water and then successively fractionated on a C18 reverse phase silica gel flash cartridge using 100 mL of each of water, methanol/water (1 : 1 v/v), and methanol, respectively. This yielded 200 mg of the standardized extract. The extract was only readily soluble in 1% ethanol (EtOH) and as such was reconstituted in it (1% EtOH) to give the various concentrations used in this study.

### 2.4. Experimental Animals

Thirty-six healthy male and female Wistar rats weighing 200–250 g were utilized in this study and were obtained from the animal house of the School of Biological and Environmental Sciences, University of Fort Hare, Alice 5700, South Africa. The animals were kept in clean polypropylene cages under standard animal house conditions (temperature: 23 ± 1°C; photoperiod: 12 h natural light and 12 h dark; humidity: 45–50%). A 7-day acclimatization period was observed before dosing and the rats were allowed* ad libitum* access to feed (Epol Feed Chunks, South Africa) and tap water under hygienic conditions. All treatments were in accordance with the* Guide for the Care and Use of Laboratory Animals* [8]. Approval (number AFO022) was granted by the Animal Ethics Committee of the University of Fort Hare, South Africa, prior to commencement of the study.

### 2.5. Acute Toxicity Test

This was performed according to the Organization for Economic Cooperation and Development (OECD) 420 guideline for testing chemicals with slight modification [9]. Twelve rats used for this study were first fasted for 18 h prior to assignment into two groups of six rats each. Each group contained equal numbers of male and female rats (i.e., 3 males and 3 females). Group A was given 1 mL single oral dose of 5000 mg/kg body weight (b.w.) of the extract, while group B (control) received 1 mL of 1% EtOH in distilled water. Following this treatment, the animals were observed closely for the first 24 h with particular attention on the first 6 h and then once daily for the 14-day experimental period. Initial and 7-day interval body weight changes of all the rats were monitored and recorded. They were also subjected to detailed gross necropsy and signs of toxicity/mortality were monitored and observed throughout the investigation period. Based on the mortality observed in each group, LD_50_ was subsequently determined [10].

### 2.6. 28-Day Repeated Dose Toxicity Test

Twenty-four Wistar strain rats were randomly divided into four groups of six animals each. Each group was made up of equal numbers of males and females. Animals in group 1 (control) were given 1 mL of 1% EtOH in distilled water. Groups 2–4 comprised animals given 1 mL of the extract at 75, 150, and 300 mg/kg b.w., respectively. All administration instances were done every 24 hours via oral gavage throughout the investigation period. The rats were weighed daily and also subjected to thorough observations for mortality, behavioral changes, and possible symptoms of humane end point during the 28-day experimental period.

### 2.7. Blood Collection and Organ Isolation

On the 29th day of the experiment, the rats were humanely sacrificed under halothane anesthesia and blood samples were collected into plain and EDTA-containing bottles as previously described [11]. The collected samples were thereafter centrifuged at 1282 ×g for 5 min using Hermie Bench Top Centrifuge (Model Hermie Z300, Hamburg, Germany) and subsequently used for biochemical and haematological analyses, respectively. The rats were immediately dissected and the liver and kidneys were isolated, freed of fat, blotted with clean tissue paper, and weighed. Relative organ-body weight ratios were thereafter evaluated.

### 2.8. Determination of Haematological Parameters

Haematological parameters, including red blood cell (RBC), haemoglobin (HGB), haematocrit (HCT), mean corpuscular volume (MCV), mean corpuscular haemoglobin (MCH), mean corpuscular haemoglobin concentration (MCHC), platelet, white blood cells (WBC), and lymphocytes, were determined using Automated Haematologic Coulter Analyzer (Beckman Coulter Inc., CA, USA).

### 2.9. Determination of Biochemical Parameters

The serum activities of aspartate aminotransferase (AST), alanine aminotransferase (ALT), alkaline phosphate (ALP), and gamma glutamyl transferase (GGT) as well as concentrations of total bilirubin, total protein, albumin, electrolytes (calcium and potassium ions), creatinine, uric acid, and urea were evaluated using Piccolo Express Automatic Chemistry Analyzer (Abaxis Inc., Union City, CA 94587, USA).

### 2.10. Histopathological Examination

A portion of each of the excised organs was fixed in 10% (v/v) buffered formaldehyde solution, dehydrated through ascending grades of ethanol (70, 90, and 95% v/v), cleaned in xylene, and embedded in paraffin wax. Tissue sections were then prepared and stained with haematoxylin-eosin [12]. The photomicrographs of the tissue sections were taken at ×400 using the Leitz DIALUX research microscope.

### 2.11. Statistical Analysis

Statistical analysis was performed using Minitab Student release version 12, Windows 95. Where necessary, data were represented as mean of six replicates ± standard error of mean and were analyzed using one-way analysis of variance (ANOVA) and complemented with Student's *t*-test. Significant differences between the treatment means were considered at *p* < 0.05.

## 3. Results

### 3.1. Acute Toxicity

The oral administration of the standardized extract of* Monsonia angustifolia* at 5000 mg/kg b.w. dose had no clinical adverse effect of substance related toxicity and did not cause mortality of any rat during the 14-day observation period. Also, there was no morbidity or behavioral or physiological changes. Compared with the control, there was no significant (*p* > 0.05) change in the body weight gain of the extract-treated rats throughout the study period (Table 1).

### 3.2. 28-Day Toxicity Test

Daily repeated oral dose treatment with the extract for 28 days did not induce any evident sign of toxicity in the treated animals, including those given 300 mg/kg b.w. dose. No deaths or obvious adverse clinical signs were observed in any of the test groups throughout the treatment period. Note that the data for both males and females in the same group were merged together for presentation in this study as there were no pronounced/significant variations in the parameters measured between the male and female rats.

### 3.3. Serum Haematological Parameters

The effects of 28-day administration of* M. angustifolia* extract at 75, 150, and 300 mg/kg b.w. doses on the haematological parameters of the animals are represented in Table 2. With the exception of the significant dose-specific increases in the plasma counts of platelets and WBC in the extract-treated rats, administration of the extract at all the investigated doses had no significant (*p* > 0.05) effect on all other parameters evaluated including HGB and RBC, when compared with the control.

### 3.4. Liver and Kidney Function Indices

Data obtained with respect to liver function parameters (AST, ALT, ALP, GGT, total bilirubin, total protein, and albumin) evaluated in this study revealed that the extract caused no significant (*p* > 0.05) alterations on these parameters for all the animals when compared with the control (Table 3). Similarly, except for the marginal variations in the serum concentrations of urea and creatinine, the extract at the tested regimens had no significant (*p* > 0.05) effect on the serum level of the electrolytes (calcium and potassium ions) and uric acid when compared with the control (Table 4).

### 3.5. Organ-Body Weight Ratio

The respective initial body weights of the treated rats and the control were compared with their final weights. Except for the significantly increased liver-body weight ratio at 300 mg/kg b.w. dose of the extract, normal body weight gains corresponding to a similar pattern of nonsignificant difference in the absolute organ weight of the kidneys and liver were observed in the extract-treated groups during the study period compared to the control group (Table 5).

### 3.6. Histopathological Examination

**Figure 1:**
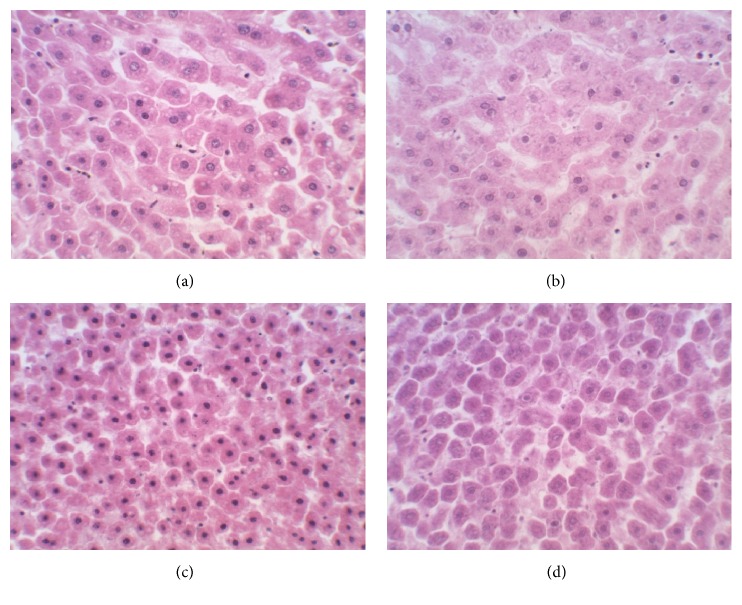
Photomicrographs (haematoxylin-eosin stained, ×400) of liver sections of Wistar rats treated with (a) 1% EtOH in water as normal control and (b) 75 mg/kg b.w. extract, (c) 150 mg/kg b.w. extract, and (d) 300 mg/kg b.w. extract of* Monsonia angustifolia* for 28 days.

**Figure 2:**
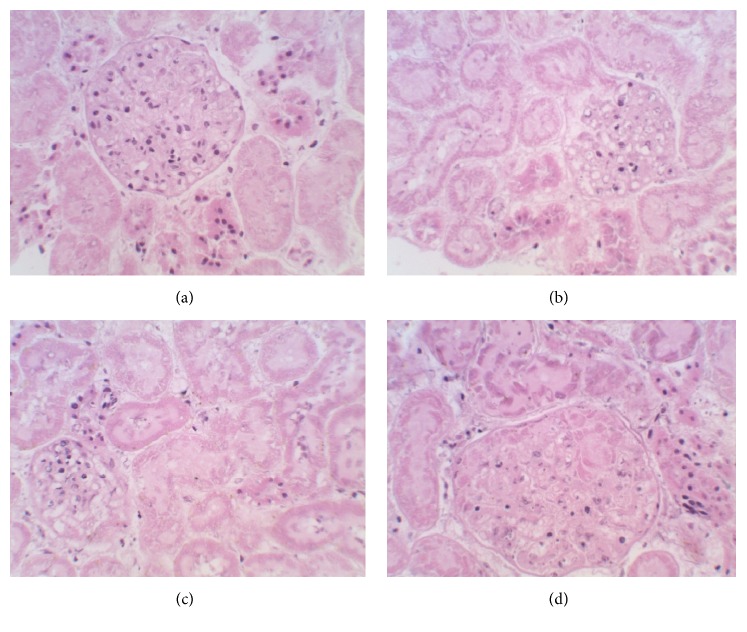
Photomicrographs (haematoxylin-eosin stained, ×400) of kidney sections of Wistar rats treated with (a) 1% EtOH in water as normal control and (b) 75 mg/kg b.w. extract, (c) 150 mg/kg b.w. extract, and (d) 300 mg/kg b.w. extract of* Monsonia angustifolia* for 28 days.

Detailed microscopic and histoarchitectural examinations of the kidney and liver of the extract-treated animals revealed no abnormalities in overall structural orientation of the organs and there were no observable cellular injuries. The nuclear characteristics, morphological features, and tissue integrity of organs of the treated rats were essentially normal and comparable to the normal control (Figures 1 and 2).

## 4. Discussion

Toxicity studies on herbal extracts are commonly used to evaluate the possible health risk of the intrinsic chemical compounds in the plant which could result in adverse effects from the plant [13]. Specifically, acute toxicity and LD_50_ determination have been described as initial steps in the toxicological evaluations of plant extracts (Oladipipo et al. [10]), and data from such evaluations provide comprehensive information on the toxicological classification and labelling of such compounds [14]. According to Lorke [15], substances with LD_50_ values of ≥5000 mg/kg b.w. are said to be safe and practically nontoxic. Hence, extract of* M. angustifolia* may be considered nontoxic when administered via oral route and could be adjudged to be relatively safe for consumption in rats. Also, that the extract at a single oral dose of 5000 mg/kg had no treatment-related adverse effect on the tested animals up to 14 days of investigation is another supportive fact of its nonacute toxicity effect. This nonacute toxicity observation could also suggest that the LD_50_ of the extract is greater than 5000 mg/kg b.w. in rats. The proportionate and nonsignificant weight gained across all the treatment groups relative to the control not only is indicative of the nontoxic potential of the extract but also suggests that growth and developmental mechanisms in the treated animals were not hampered.

Since treatment-related toxicity was not evidenced during the acute toxicity evaluation, further testing was conducted to evaluate the 28-day repeated daily dose of the extract on key metabolic markers of rats. This was done with a view to providing comprehensive toxicological data on this emerging botanical. The selected doses (75, 150, and 300 mg/kg b.w.) in this study were informed by the averages of daily consumed regimens and reported pharmacological study on the extract [6]. Generally, the fact that 28-day daily dose treatment with the extract elicited no clinical signs of toxicity, morbidity, or mortality across all the treatment groups may be a tenable inference that the extract of* M. angustifolia* is unlikely to be toxic at the tested doses over the observation period.

Investigation on the haematological parameters can be used to determine the extent of the deleterious effect of foreign compounds in plant extracts on the blood constituents of an animal [16]. In this study, the nonsignificant difference in RBC and HGB counts following repeated daily dose treatment with the extract could be an indication that it may not be toxic to the blood. This implies that the morphology and osmotic fragility of the RBC, as well as HGB incorporation into the RBC, were not affected. This may also suggest that the oxygen-carrying capacity of the blood and amount of oxygen delivered to the tissues following treatment with the extract are intact [17]. Evaluation of indices (HCT, MCV, MCH, MCHC, and RCDW) relating to the status of RBC is imperative to the diagnosis of anaemia in animals [17]. The nonsignificant effect on these indices for the extract-treated animals relative to control may be an indication that the extract at the tested doses had no overall adverse effect on RBCs' microcytes and HGB weight per RBC. This suggests that the 28-day daily oral dose treatment with the extract does not predispose the animals to anaemic condition. Our submission is consistent with the findings of Ashafa and Olunu [18], where the administration of ethanolic root extract of* M. lucida* was nonhaematotoxic to the animals. The plasma level of the WBC counts is a pointer to an organism's defensive potential against infections. The dose-dependent significantly increased WBC counts following* M. angustifolia* extract administration in the animals may indicate immune system boost. The increase in the white blood cells could also suggest that the effects on cells of the immune system at the tested doses were not adversely affected and further supported the nonhaematotoxic nature of the extract. This agrees with the report of Ashafa and Kazeem [19], who gave similar submission on administration of* Dianthus basuticus* on leukocyte status of rats. That the extract also dose-dependently increased platelet counts in the animals over the 28-day investigation period may be informative of its stimulatory effect on thrombopoietin. This is not only suggestive of the extract's capability to considerably manage thrombocytopenia in rats (Geddis [20]), but also indicative of its unlikely toxicity.

Liver and kidney function tests are crucial in toxicological evaluation of plant extracts due to the utmost involvement of these organs in xenobiotic biotransformation [6]. Significantly increased serum activities of ALP, ALT, AST, and GGT are closely associated with hepatic injury [21]. The nonsignificant differences in the serum activities of these marker enzymes in the extract-treated rats relative to normal control are informative either of the fact that the extract does not impede hepatocytes function in the rats or of the fact that the integrity of the liver cells was not compromised. Additionally, concentrations of total protein, bilirubin, and albumin in the serum may indicate the state of the liver and the type of damage. The nonsignificant effect of the extract on these parameters further confirms that it is unlikely to be hepatotoxic. Kidney damage may be ascertained by measurements of urea, uric acid, creatinine, and electrolytes, and deviations from normal in their serum concentrations are a tentative pointer to nephrotic injury [22]. In this study, the nonsignificant difference observed in the kidney function indices in the extract-treated animals is suggestive of normal renal function and further supports the nontoxic tendency of the extract.

A drastic change in body weight is a critical evaluator of toxicity and may serve as a sensitive indication of the general wellbeing of animals [23]. The mean body weight gained by the animals in all the treatment groups may be an indication that the extract did not interfere with their normal metabolism as closely supported by the nonsignificant difference in this parameter when compared with the control group. The increase in body weight could be attributed to the nutritive components in their feed and the palatability of the extract [24]. Organ-body weight ratio may indicate organ swelling, atrophy, or hypertrophy [25]. In this study, the nonsignificant changes in the weights of the liver and kidneys suggest that these organs neither were adversely affected nor produced treatment-related/clinical signs of toxicity throughout the treatment period with the extract. However, the observed increase in the absolute liver-body weight ratio in the rats given 300 mg/kg b.w. dose of the extract could be regarded as being toxicologically and biochemically insignificant as it was not corroborated by both the histological examination and other clinical biochemical parameters of liver function evaluated in this study.

Besides complementing biochemical investigations, histological examination of organs following exposure to pharmacological agents is an important consideration in assessing the safety of such agents on organ injury [17]. Hence, the apparently preserved histoarchitectural features as evident from microscopic examinations of the kidneys and liver sections of the extract-treated animals in this study are another supportive fact that the organs were void of injuries and further indicate that* M. angustifolia* extract was not toxic to them at the tested doses. Furthermore, the no treatment-induced infiltration and inflammation as shown in the microscopic examination of the organs from extract-treated groups are also supportive of its capability to maintain and sustain histoarchitectural integrity of the organs. In light of the foregoing, it may be inferred that the extract of* M. angustifolia* is unlikely to be toxic (at the investigated doses) to the studied organs in this study and also supported its pharmacological significance.

## 5. Conclusion

Overall, it is evident from the present study that LD_50_ of the standardized extract of* M. angustifolia* is well above 5000 mg/kg b.w. in Wistar rats. Following its 28-day repeated daily oral dose administration in the animals, it may be concluded that it does not elicit any treatment-related adverse effect at the doses investigated and thus may be classified to be relatively safe and practically nontoxic for consumption. Although work is in progress in evaluating its safety tendency on other systemic organs and tissues in animals, the data from this study have supported the safety and therapeutic importance of* M. angustifolia* in the traditional system of medicine.

## Figures and Tables

**Table 1:** Body weight changes of the animals following single oral dose administration of 5000 mg/kg body weight of *Monsonia angustifolia *extract.

Groups	Weekly weight changes (g)			% weight gain
	Day 0	Day 7	Day 14	
Control (distilled water)	226.08 ± 1.89	237.10 ± 1.39	243.48 ± 1.16	7.15
Extract (5000 mg/kg)	231.24 ± 1.45	245.32 ± 1.11	248.55 ± 1.43	6.97

Values are means ± SEM (*n* = 6). Test values not sharing a superscript are nonsignificantly different from the control (*p* > 0.05).

**Table 2:** Effect of 28-day administration of standardized extract of *Monsonia angustifolia *on haematological parameters of Wistar rats.

Parameters	Control	Extract (mg/kg body weight)		
		75	150	300
HGB (g/dL)	15.30 ± 0.62	15.67 ± 1.75	15.63 ± 1.05	15.23 ± 1.36
Haematocrit (L/L)	0.46 ± 0.02	0.49 ± 0.36	0.48 ± 0.02	0.45 ± 0.01
MCV (fl)	59.20 ± 0.69	58.93 ± 1.46	60.97 ± 1.66	62.03 ± 1.45
MCH (pg)	18.77 ± 0.25	18.67 ± 0.93	18.83 ± 0.40	19.03 ± 0.65
MCHC (g/dL)	31.6 ± 0.69	31.93 ± 0.75	31.23 ± 1.22	30.13 ± 0.38
RCDW (%)	12.67 ± 0.31	12.70 ± 0.46	12.83 ± 0.40	13.07 ± 0.49
RBC (10^12^/L)	7.67 ± 0.37	8.31 ± 0.46	8.22 ± 1.21	8.01 ± 0.88
Platelets (10^9^/L)	889.7 ± 1.15	854.0 ± 1.53	955.3 ± 1.00^a^	1010.0 ± 1.41^b^
MPV	7.90 ± 0.10	8.00 ± 0.36	7.60 ± 0.10	7.57 ± 0.40
WBC (10^9^/L)	13.44 ± 1.04	17.42 ± 0.99^a^	20.42 ± 0.48^b^	24.49 ± 0.53^c^
Neutrophils (10^9^/L)	10.67 ± 0.25	10.97 ± 0.89	10.18 ± 0.21	9.53 ± 0.66
Monocytes (10^9^/L)	57.00 ± 1.83	56.05 ± 1.77	54.75 ± 0.21	55.50 ± 1.82
Eosinophils (10^9^/L)	2.47 ± 0.72	2.23 ± 0.39	2.33 ± 0.41	2.50 ± 0.91
Basophils (10^9^/L)	0.60 ± 0.26	0.40 ± 030	0.53 ± 0.21	0.57 ± 0.51
Lymphocytes	25.47 ± 0.02	26.40 ± 1.41	26.80 ± 3.25	27.73 ± 0.99

Data are means ± SEM (*n* = 6). Values carrying superscripts are significantly different (*p* < 0.05) from the control. RBC: red blood cell; HGB: haemoglobin; MCV: mean corpuscular volume; MCH: mean corpuscular haemoglobin; MCHC: mean corpuscular haemoglobin concentration; RCDW: red cell distribution width; MPV: mean platelet volume; WBC: white blood cell.

**Table 3:** Effect of 28-day administration of *Monsonia angustifolia *extract on some liver function parameters of Wistar rats.

Parameters	Control	Extract (mg/kg body weight)		
		75	150	300
Albumin (g/dL)	16.67 ± 0.58	16.33 ± 0.06	17.07 ± 0.58	17.01 ± 0.58
Total protein (g/dL)	56.67 ± 1.51	59.67 ± 1.53	58.67 ± 1.51	57.33 ± 1.51
Total bilirubin (*μ*mol/L)	7.00 ± 0.65	7.32 ± 0.00	7.37 ± 0.08	7.50 ± 0.54
AST (U/L)	140.33 ± 1.79	139.33 ± 1.15	141.33 ± 1.08	144.65 ± 1.71
ALT (U/L)	61.67 ± 1.02	62.67 ± 1.29	63.00 ± 1.44	63.67 ± 1.72
ALP (U/L)	380.30 ± 1.53	382.30 ± 1.15	385.00 ± 1.58	383.30 ± 1.00
GGT (U/L)	1.67 ± 0.53	1.70 ± 0.00	1.70 ± 0.00	1.67 ± 0.15

Data are means ± SEM (*n* = 6). Values not carrying superscripts are not significantly different (*p* > 0.05) from the control. AST: aspartate aminotransferase; ALP: alkaline phosphatase; ALT: alanine aminotransferase; GGT: gamma glutamyl transferase.

**Table 4:** Effect of 28-day administration of *Monsonia angustifolia *extract on some kidney function parameters of Wistar rats.

Parameters	Control	Extract (mg/kg body weight)		
		75	150	300
Creatinine (mg/dL)	28.00 ± 0.71	30.00 ± 2.83	29.00 ± 3.61	28.67 ± 2.52
Uric acid (mg/dL)	5.33 ± 0.15	5.43 ± 0.35	5.56 ± 0.31	5.70 ± 0.10
Urea (mg/dL)	7.33 ± 1.12	6.90 ± 2.59	6.83 ± 0.98	7.17 ± 0.76
Calcium (mg/dL)	8.13 ± 0.06	8.36 ± 0.21	8.33 ± 0.25	8.16 ± 0.12
Potassium (mmol/L)	5.20 ± 0.25	5.53 ± 3.14	5.20 ± 0.96	5.37 ± 0.21

Data are means ± SEM (*n* = 6). Values not carrying superscripts are not significantly different (*p* > 0.05) from the control.

**Table 5:** Effect of 28-day administration of *Monsonia angustifolia *extract on the body and organ weights of rats.

Parameters	Control	Extract (mg/kg body weight)		
		75	150	300
Initial body weight (g)	283.52 ± 0.03	282.41 ± 0.19	283.33 ± 2.74	284.58 ± 2.87
Final body weight (g)	311.94 ± 0.06	322.28 ± 0.19	318.21 ± 0.86	333.11 ± 0.86
Weight of liver (g)	11.97 ± 0.20	12.44 ± 0.05	12.06 ± 0.36	14.99 ± 0.26
Weight of kidney (g)	2.33 ± 0.20	2.56 ± 0.06	2.46 ± 0.09	2.35 ± 0.06
Liver-body weight ratio (%)	3.84 ± 0.03	3.86 ± 0.06	3.79 ± 0.21	4.50 ± 0.01^a^
Kidney-body weight ratio (%)	0.75 ± 0.02	0.80 ± 0.02	0.82 ± 0.03	0.78 ± 0.01

Data are means ± SEM (*n* = 6). Values carrying superscripts are significantly different (*p* < 0.05) from the control.
